# Q fever abortions in ruminants and associated on-farm risk factors in northern Cyprus

**DOI:** 10.1186/1746-6148-7-13

**Published:** 2011-03-17

**Authors:** Leon Cantas, Adrian Muwonge, Baris Sareyyupoglu, Hakan Yardimci, Eystein Skjerve

**Affiliations:** 1 Department of Food Safety and Infection Biology, Norwegian School of Veterinary Science, P.O. Box 8146 Dep., 0033 Oslo, Norway; 2 Department of Microbiology, Faculty of Veterinary Medicine, Ankara University, Diskapi, 06110 Ankara, Turkey; 3 Centre for Epidemiology and Biostatistics, Norwegian School of Veterinary Science, P.O. Box 8146 Dep., 0033 Oslo, Norway

## Background

Q fever is caused by the obligate intracellular bacterium, *Coxiella burnetii *[1,2]. This disease is regarded as endemic worldwide, with the exception of New Zealand [3-6]. Cattle, sheep and goats are considered to be the primary source of transmission for humans [7,8]. Humans are infected mainly by inhalation of contaminated aerosols or by the ingestion of infected milk and/or fresh dairy products. In animals, Q fever is mainly subclinical but has especially been associated with reproductive disorders such as late abortions, stillbirths, weak off springs, metritis and infertility in ruminants [8-11]. Abortions during Q fever epizootics have been described in goats and sheep, but rarely documented in dairy cows [7,12].

Domestic pets, such as cats, dogs and wild-domestic birds such as rock doves (*Columba livia*) and geese (*Anser anser*) are known to be an additional source of infection [8,12-14]. Previous studies have reported occurrences of *C. burnetii *in migratory wild birds, rodents and ticks in southern Cyprus [15-17]. More than 40 species of ticks are naturally infected with *C. burnetii*. However, besides the aerosol route, the significance of ticks in transmitting the disease in ruminants and humans has previously been documented [8,9]. On the other hand, recent studies showed that ticks seem to play a major role in the circulation of *C. burnetii *in cycles of nature especially in wild life cycles. Ticks are also believed to probably play another crucial role in the transmission of the agent from infected wild vertebrates to domestic animals [5,18,19].

In humans, Q fever is mostly asymptomatic, the acute disease form is mainly limited flu-like illness, pneumonia or hepatitis while the chronic disease manifests with chronic fatigue syndrome or endocarditis [4,5,20]. On the reproductive health point of view, *C. burnetii *infections are known to cause abortions, stillbirth and pre-mature deliveries in pregnant women. In the past, a series of Q fever outbreaks in both human and animal populations resulting in abortions on the island of Cyprus have been reported [10,21]. Studies done on the islands as far back as the 1970's showed that Q fever has been an ongoing public health problem. Recently, the prevalence of IgG antibodies against *C. burnetii *phase II antigens was estimated to be at 52.7% for humans, 48.2% for goats, 18.9% for sheep, and 24% for cows. In this context, control of *C. burnetii *infection in ruminants is a vital component of public health [19]. There is no known record of humans contracting Q fever in northern Cyprus, which might be linked with lack of routine screenings and/or insufficient diagnostic units for *C. burnetii *[Northern Cyprus Ministry of Health, 2008].

The diagnostic enigma is that *C. burnetii *is difficult to culture, and detection in Cyprus was first done by complement fixation of antibodies [22,23]. Lately detection and diagnosis of *C. burnetii*, has been more effectively done by PCR based techniques, targeting the isocitrate dehydrogenase, superoxide dismutase gene and a transposon-like repetitive region [15,24-28]. The current technological advancement in these techniques has made them the most useful diagnostic tools for detection of *C. burnetii *in bovine aborted foetuses and ovine genital swabs [29-31].

Since the division of Cyprus in 1974, there has not been any research work on this disease in the northern region. Therefore, the aim of this study was to determine the occurrence of Q fever abortion using a PCR based method on DNA isolated from aborted foetal abomasal contents and placental tissues from ruminants in northern Cyprus. In addition, to determine the on-farm risk factors associated with the disease.

## Methods

### Study area

Northern Cyprus covers about 37% of the third largest island in the Eastern Mediterranean, located south of Turkey and west of Syria and Lebanon and is divided into three main regions; Northbound Region (Kyrenia and North of Nicosia), Border Region (Morphou, South of Nicosia, Famagusta and Vadili) and Karpas Region (Gecitkale, Iskele). It has a population of 265.100 people [Governmental Planning Office-Northern Cyprus 2006]. The economy is dominated by the service sector, but the animal husbandry industry is growing steadily. The region has a ruminant population of approximately 50.000 bovine of which 18.000 are milking cows, 185.000 are sheep and 45.000 are goats [Northern Cyprus Veterinary Service 2008]. Climatically, summers are dry and hot while winters are mild. The average annual temperature and rainfall is 19°C and 345 mm respectively. The dairy based regions are located at sea level and cattle raring is more intensive than semi-extensive for small ruminant farms. Generally milking cow farms are urged to report any cases of abortions in order to be compensated for the economical loses. However, this policy does not apply to small ruminant farms [Northern Cyprus Veterinary Service 2008]

### Study design and sampling strategy

This study was based on a cross-sectional convenient sampling strategy. The government veterinary services received a total of 622 reports of third trimester cow abortion cases from the entire northern region in the period between October 2008 and March 2009. Owing to long distances between the farms, lack of coordination between farmers and veterinary services, financial logistical problems, only 51 different milking cow case-farms were conveniently visited and abortion materials were sampled from 51 different cows. As small ruminant farmers were not urged to declare abortions, only eight different small ruminant abortions/farms (6 sheep and 2 goats) could be included into this study. Abomasal content and a piece of cotyledon were collected from each abortion case, therefore a total 59 abortion case materials were collected in duplicates (abomasal content and cotyledon). A questionnaire was also administered to each visited farm. Information regarding; geographical location of farm, type of sampled animal, and type of animal feed, cleaning frequency of barn floor, presence of carnivores, pigeons on the farm and the presence of ticks on aborted animal was gathered. In addition, weather information was also collected from the meteorological centre in northern Cyprus during the study period.

### Sample collection and laboratory analysis

Sterile latex gloves and face masks (that adequately cover the mouth and nose) were used during the sample collection procedures. A piece of cotyledon sample (20 g) was carefully cut from the placenta and placed in a sterile labeled collection container, thereafter the fetus was dissected to expose the abomasum from which abomasal contents (5-10 ml) were collected and placed in a separate sterile tubes. Gloves were changed between each collection to avoid cross contamination. The samples were then transported in a cold box to the laboratory in Ankara.

DNA was extracted from foetal abomasal contents and placental cotyledon using the DNeasy Blood & Tissue Kit^® ^(Qiagen S.A., France). The positive ready to use control DNA of *C. burnetii *Nine Mile phase II strain was provided by Dr. Amanda Loftis Rickettsial Zoonoses Branch, Centers for Disease Control and Prevention, Atlanta, USA.

Two different PCR reactions (CB-PCR and Trans-PCR) were run as described below:

Initially, a 257 base pair (bp) fragment was amplified by CB-PCR to detect superoxide dismutase gene with CB-1 and CB-2 primers [32]. Thereafter, a 687 bp fragment was amplified by Trans-PCR to detect IS1111A transposase gene with Trans-1 and Trans-2 primers [28]. These two PCR reactions were performed in 25 μl reaction mixture containing 2.5 μl template DNA, 2.5 μl 10xPCR Buffer, 3 μl 25 mM MgCl_2, _0.5 μl 10 mM dNTP mix, 5 pMol each of forward and reverse primers, 1U of Taq DNA polymerase (Fermentas, Vilnius, Lithuania) and 15.4 μl sterile nuclease-free PCR grade water. In order to avoid cross contamination the PCR mixture was prepared in laminar flow cabinet equipped with a UV lamp, in a separate room. During the process fresh gloves were used and pipette tips with aerosol filters were preferred. DNA amplifications were performed in a T1 Thermocycler (Biometra, Germany). Positive and negative control (ultra pure water) samples were included in all amplifications. PCR conditions and amplification cycles were identical with previously described original protocols [26,28]. PCR assay specificity was tested with the amplification protocols described above using DNA extracted from all field and reference *C. burnetii *Nine Mile phase II strain as a template that has a known concentration (65 ng/ml) of ready to use DNA. The resultant PCR products were analyzed on 1.5% agarose gel. After electrophoresis at 100V for 60 min, gels were stained with ethidium bromide and visualized by a Bio Imaging System (Syngene, Cambridge, UK).

### Data analysis

A positive animal was defined as one that was positive on both tests on DNA isolated from abomasal content, therefore this served as the gold standard for this study. The on farm based variables like source of feed for animals, frequency of litter cleaning, presence of ticks on aborted animals, pigeons, rodents and carnivores on the farm were coded and together with the corresponding PCR based test results from each farm were entered in a Microsoft Excel^® ^spreadsheet. After validation the data was then transferred to Stata (stata/SE 10 for Windows, StataCorp, College Station,TX) for statistical analysis. A survey-data-analysis procedure was used for estimating the occurrence of Q fever. The univariable association of Q fever (Odds ratio with 95% CI) with individual exposure variables, considering individual animal as primary sampling unit was determined. A p ≥ 0.25 was used as a cut off value for exposure factors that were included in the univariable analysis, a Multivariable logistic regression model analysis was then built with forward selection procedure to determine the risk factors. Model validity and reliability was assessed using the Hosmer-Lemeshow goodness of-fit test and receiver operating curve (ROC) respectively. In addition the degree of agreement between the two PCR diagnostic methods on each sample was analyzed using the Kappa agreement measure using SISA http://www.quantitativeskills.com/sisa/statistics/diagnos.htm online based software.

## Results

Of the 59 sampled ruminants, twenty two (37%) were positive for both PCR based tests on foetal abomasal content used in this study (Table 1). 35% (18/51) of bovine, 33% (2/6) of sheep and 50% (1/2) of goat abortion cases were positive for *C. burnetii *with the Trans and CB PCRs test foetal abomasal content. Nineteen (%32.2) of uterine cotyledon DNA were positive for both PCR test (Table 2). The diagnostic agreement of the tests is compared in Table 1 and Table 2. Trans-PCR and CB-PCR assay had a kappa diagnostic agreement of 64% and 68% on foetal abomasal content and uterine cotyledon respectively.

**Table 1:** Diagnostic agreement between CB-PCR and Trans-PCR assay on DNA isolated from abomasal content.

Cows _(51) _- Sheep _(6) _- Goat _(2) _Abortions				
	**Trans-PCR**			
		Positive	Negative	Total
	Positive	22	5	27
**CB-PCR**	Negative	7	35	32
	Total	29	40	59
		Kappa agreement between CB and Trans PCR 64% (34-94) SE 12%		

**Table 2:** Diagnostic agreement between CB-PCR and Trans-PCR assay on DNA isolated from placental cotyledons.

Cows _(51) _- Sheep _(6) _- Goat _(2) _Abortions				
	**Trans-PCR**			
		Positive	Negative	Total
	Positive	19	6	25
**CB-PCR**	Negative	4	40	44
	Total	23	46	59
		Kappa agreement between CB and Trans PCR 68%(38-98) SE 12%		

Table 3 shows geographical distribution in the occurrence of *C. burnetii *abortion among ruminants, 35%, 53% and 42% of the reported sample abortion cases in the Northbound Region, Border Region and Karpas Region respectively were caused by *C. burnetii*. The univariable analysis shows that farms which used commercially produced feed and cleaned litter more than 10 times in a year were less likely to have abortions due to *C. burnetii *compared to those using farm-made feed and clean litter <5 times in a year (Table 3). The logistic regression model identified ticks (OR = 4; P = 0.05), poor hygiene (OR = 0.3; P = 0.05 and OR = 0.09; P = 0.05) and presence of carnivores (OR = 3; P = 0.01) as the on-farm risk factors associated with occurrence of *C. burnetii *abortions (Table 4). The logistic regression model fits the data (HL χ2 = 3.01; P = 0.69) and the evaluation of reliability showed that it was reliable (ROC = 0.84 and 0.79).

**Table 3:** Univariate association between recorded exposure factors and prevalence of Q fever abortions in ruminants.

Variable	Label	No of examined animals	PCR positive for *C. burnetii*	p-value
**Regions**	Northbound Region	13	7	-
	
	Border Region	26	11	0.50
	
	Karpas Region	20	7	0.30

**Animals**	Cows	51	21	-
	
	Sheep and Goat	8	4	0.64

**Feed Type**	Farm-made Feed	28	19	-
	Commercial Feed	23	3	0.00
	
	Both of The Above	8	3	0.13

**Frequency of Litter Cleaning**	<5 Times/Year	23	19	-
	
	5 < × < 10 Times/Year	20	5	0.001
	
	>10 Times/Years	16	1	0.001

**Presence of Rodents in Animal Housing**	Absent	33	9	-
	
	Present	26	16	0.01

**Ticks on aborted Animals**	Absent	29	2	-
	
	Present	30	23	0.001

**Houseflies on Farm**	Absent	23	4	-
	
	Present	36	21	0.003

**Pigeons on Farm**	Absent	24	3	-
	
	Present	35	22	0.001

**Presence of Carnivores at Farm**	Absent	30	4	0.001
	
	Present	29	21	0.001

**Table 4:** Multivariable logistic regression model for risk factors associated with Q fever abortion in ruminants.

Variables	Level	Odds ratio	P-value	95% CI
Ticks on aborted animals	Absent	1.00	-	-
	Present	4.54	0.05	0.96-21.32
Frequency of litter cleaning	<5 times/year	1.00	-	-
	5 < × < 10 times/year	0.30	0.05	0.04-1.00
	>10 times/year	0.09	0.05	0.008-1.023
Carnivores on Farm	Absent	1.00	-	-
	Present	3.33	0.01	0.762-1.02

**Figure 1:**
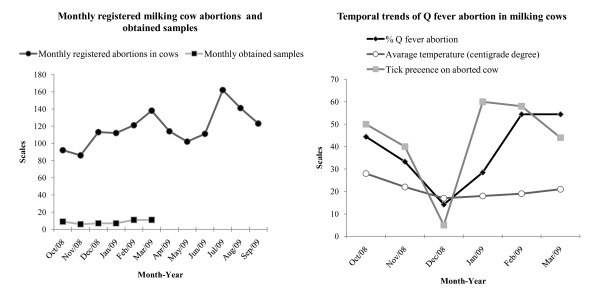
**Monthly registered cow abortions (October 2008-September 2009) and temporal trends of *C. burnetii *related cow abortions (October 2008-March 2009) in northern Cyprus**.

The veterinary service data base had a total of 1415 abortions among ruminants between October 2008 and September 2009. However, 662 of these were reported during the time of study. The lowest registered number of abortion cases in the general data base was in November while the lowest occurrence of *C. burnetii *abortions was in December. However, this occurrence gradually increased from January to another peak in February and then decreased towards March. The *C. burnetii *abortions and presence of ticks on abortion cow cases seem to follow the gradual fall in temperature as the season transition from autumn to winter (Figure 1).

## Discussion

To the best of our knowledge, this is the only documented epidemiological study on Q fever in ruminants in northern Cyprus since 1974. From a total of 59 randomly sampled abortion cases in ruminants, 22 (37%) were found to be caused by *C. burnetii*. This is higher than the prevalence documented in Italian domestic ruminant abortions (22.35%) [33]. Border Region (53%, 11/26) had the highest occurrence of *C. burnetii *related abortions (Table 3). This region is mostly a rural area and based on the questionnaire, poor hygiene practices may explain the relatively high occurrence of *C. burnetii *abortions. Furthermore, in a previous larger study, Psaroulaki et al. 2006 showed that the border region had the highest cumulative sero-prevalance rates of *C. burnetii *specific antibodies in small ruminants, in southern Cyprus. Although animal flocks have not been in close contact among the border line since 1974, in epidemiological frame-work [4,6] uncontrolled *C. burnetii *in northern part (particularly in Border region) may play a key role in the circulation of the problem between two areas.

*C. burnetii *is a fastidious intracellular bacterium whose isolation requires several days to weeks involving a difficult and yet hazardous procedure. This is in addition to the heterogeneity in growth conditions required by the different strains of *C. burnetii *[34]. Therefore, the international office of epizootics (O.I.E) recommends PCR as one of the most effective methods for *C. burnetii *diagnosis. Trans-PCR and CB-PCR assay had a diagnostic agreement measure of 64% and 68% on foetal abomasal contents and uterine cotyledon respectively (Table 1 and Table 2). This disparity could be due to the fact that placental tissues are prone to contamination from faecal material during abortion, which is known to contain inhibitors to Taq polymerase [35]. These inhibitors can therefore interfere with the PCR procedure and thus affect the results. Anti-inhibitors of Qiamp Tissue DNA purification procedure were used but it is possible that did not completely eliminate PCR inhibitors from placental cotyledons. This finding is in agreement with the study by [29]. Therefore animals were deemed positive if they were found positive for both tests on DNA from foetal abomasal content based on this fact. This study therefore found that a combination of Trans PCR and CB-PCR assay on foetal abomasal content gave a better diagnostic edge.

This study also found tick presence on aborted animals (OR = 4; P = 0.05) as the most plausible on farm management risk factor with regards to Q fever occurrence in northern Cyprus (Table 4). It has been documented that in nature C*. burnetii *is found primarily in a cycle where ticks play a significant role in transmitting the pathogen among wild vertebrates, such as rodents, lagomorphs, and wild birds [8,18,36]. This therefore concurs with studies done before showing the key role ticks play as transmitting agents of *C. burnetii *between wild life and livestock [6,8]. On the other hand, carnivore (dogs and cats) presence on the farm also presented an additional risk (OR = 3; p = 0.01) however, like the rodents these seem to fit in tick transmission cycle as elaborated in studies done by [9,18].

Good hygiene practices are an important way of reducing the risk of spread of infectious diseases, and the findings of this study agree with this notion. The higher the frequency of litter cleaning (5 < × < 10 and × > 10 times/year) on farm the more protective (OR = 0.3; P = 0.05 and OR = 0.09; P = 0.05) it was against the risk of Q fever (Table 4). *C. burnetii *is a pathogen known to be resistant to disinfectants and environmental factors, and Brouqui et al. 2007 reported that *C. burnetii *could survive for years in animal faeces for 8-9 months in sand and 19 months in dried tick faeces. Studies done in rural areas have all indicated that poor hygiene could be an exacerbating factor in the spread of *C. burnetii *[37,38].

This study also found a seasonal variation in the occurrence of *C. burnetii *abortions in northern Cyprus (Figure 1). The highest occurrence was experienced in October which gradually declined to the lowest in December. This trend follows the steady fall of temperatures that characterizing the transition from autumn to winter. This change of season could have a direct effect on tick prevalence which then indirectly affects the occurrence of the disease in this region. Unfortunately this study did not examine the tick species on abortion ruminant cases, but previous studies showed that more than 40 species are naturally infected and play role in transmission of the agent [8,9]. Therefore regardless of the tick species, presence of ticks on farm is a risk given the wide tick species range that carry C*. burnetii*. The seasonal variation of C*. burnetii *has previously been documented in cattle in Japan [39], however, this study documented in sero-prevalance rates. On the other hand in humans, seasonality has been seen to occur in spring following the outdoor lambing of sheep in southern Germany [40]. Unfortunately, no data are yet available on the occurrence of human Q fever in northern Cyprus. The findings of our study suggest that more investigations are necessary on this largely underestimated public health issue in northern Cyprus.

Human disease contracted from livestock is already a public health problem in southern Cyprus [41]. The identified risk factors in this study can be used to avert a potentially catastrophic public health problems in northern Cyprus in the following ways; i) on the farm level, a stringent tick control strategy is very important in breaking the spreading cycle to cattle, carnivores, rodents and pigeons. In addition rodent free housing is paramount in removing the extra circulating agents. Hygiene on farm should be a routine management practice, ii) at National level besides milking cow abortions, small ruminant farms also should be urged to declare abortion cases, it is important to put in place a vigorous surveillance system to keep track of outbreaks and occurrences given the endemic nature of the infection. This ought to be in addition to a strategic vaccination scheme, iii) on a regional level, given the differences in disease occurrence between northern and southern Cyprus. There should be a strict control of animal movement between the two regions and a joint concerted effort with regard to disease control and research on regional based vaccine development.

## Conclusions

In conclusion, this study found a significantly high occurrence of *C. burnetii *abortions in northern Cyprus. Ticks on aborted animals and poor hygiene were identified as the most important on-farm risk factors associated with *C. burnetii *abortions. In additional Trans-PCR and CB-PCR assay on foetal abomasal content was a better diagnostic combination.

## Authors' contributions

LC contributed to the establishment of the collaborations, design, conception, data collection, performed the all laboratory work, data analysis, drafting and writing of the manuscript. AM contributed data analysis, drafting and writing of the manuscript. BS contributed to laboratory analysis, design and supervision of the manuscript. HY contributed to the acquisition of funds, drafting of the manuscript. ES contribute to the supervision, drafting and writing of the manuscript. All authors contributed to the final version of the manuscript, read and approved it.
